# Intrabiliary Hepatic Metastasis of Colorectal Carcinoma Mimicking Primary Cholangiocarcinoma: A Case Report and Review of the Literature

**DOI:** 10.1155/2016/4704781

**Published:** 2016-06-26

**Authors:** Yimin Dong, Hitendra Patel, Charmi Patel

**Affiliations:** ^1^Department of Pathology, School of Medicine, University of Arizona, Tucson, AZ 85724, USA; ^2^Division of Hematology Oncology, Department of Medicine, School of Medicine, University of Arizona, Tucson, AZ 85724, USA

## Abstract

Intrabiliary metastasis from colorectal carcinoma (CRC) growing within or invading bile ducts is not a very common pattern. However, accurate diagnosis of metastatic lesions is very important for selection of adjuvant therapy and prognosis. We report a case of 71-year-old male who developed painless jaundice due to hepatobiliary obstruction. MRI demonstrated 1.4 cm intraductal mass at hepatic hilum with severe intrahepatic ductal dilation, consistent with cholangiocarcinoma. ERCP (endoscopic retrograde cholangiopancreatography) showed intraductal segmental biliary stricture. Biopsy from the lesion showed adenocarcinoma favoring primary cholangiocarcinoma due to the papillary morphology and location of the mass. His past history was significant for rectosigmoid carcinoma (pT1N0) ten years ago and liver resection for metastatic CRC four years ago. He subsequently underwent central hepatectomy with resection of common bile duct. Grossly, there was a 1.2 cm intraductal mass at the bifurcation of bile ducts with multiple nodules in liver parenchyma. Microscopic examination revealed intraductal carcinoma with papillary architecture colonizing bile duct epithelium with resultant dilation and tortuosity. Occasional liver parenchymal nodules show classical metastatic pattern resembling CRC. Because of two distinct morphologic patterns and patient's past history, immunostains were performed. CK7 stained uninvolved bile duct epithelium with no staining in intrabiliary metastatic growth. CK20 and CDX2 were positive, thus confirming intrabiliary growth as metastatic growth from CRC. In summary, findings from our case indicate that intrabiliary growth of metastatic CRC can easily be overlooked with major duct involvement. Pathologic evaluation with use of immunohistochemical stains is very important to achieve correct diagnosis.

## 1. Introduction

Intrabiliary growth along the biliary track is not a well-recognized behavior for hepatic metastasis. Biliary involvement by colorectal liver metastases (CRLM), also known as intrabiliary metastasis of colorectal carcinoma, is a clinically rare event, with the annual incidence of 0.00067% in the United States [1]. There have been only two statistical studies for these metastases in the western population regarding the survival and prevalence [2, 3]. This metastasis is an occult process that the average interval from the primary lesion resection to intrahepatic metastatic lesion resection is approximately 28 months [2]. Moreover, it is difficult to differentiate intrabiliary CRLM from primary intrahepatic cholangiocarcinoma (PICC) morphologically [4, 5]. Dysplastic change in biliary epithelium is used to differentiate primary cholangiocarcinoma from metastatic lesions. Therefore, careful evaluation of patient's history and morphologic features with immunohistochemical (IHC) studies, such as CK7, CK20, and CDX2 staining, can provide definite diagnosis. Here, we reported a case of intrabiliary CRLM that happened approximately 10 years after the resection of the primary rectosigmoid carcinoma and discussed the characteristics of intrabiliary metastasis and its differentiations from the PICC.

## 2. Case Presentation

**Figure 1:**
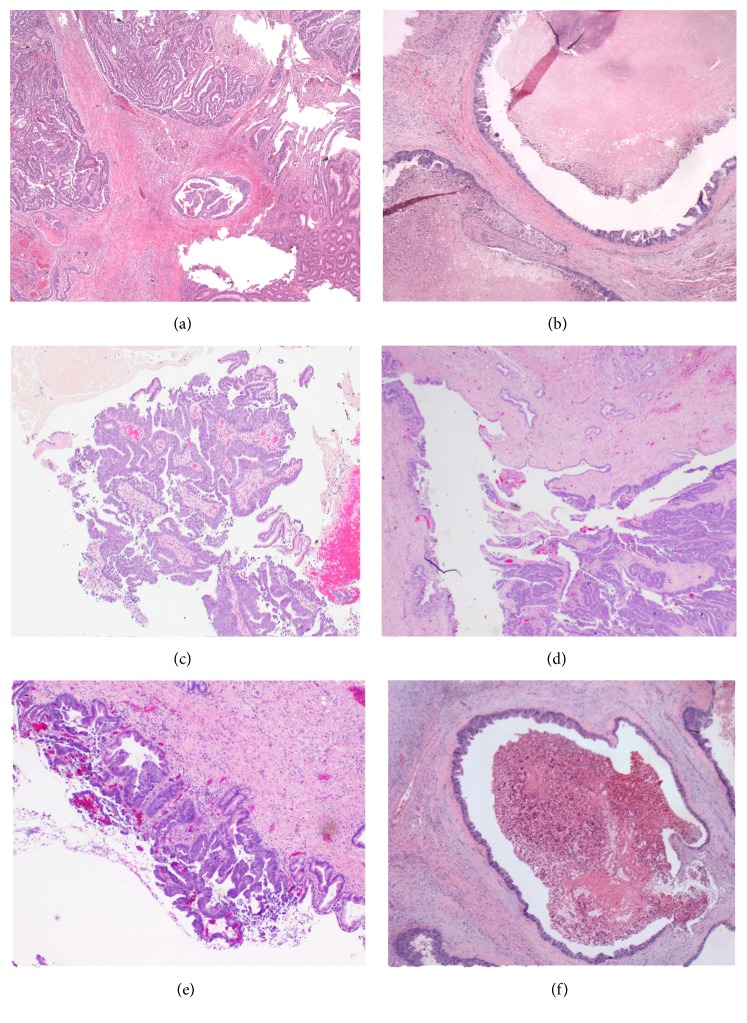
(a) Patient's primary rectosigmoid adenocarcinoma. (b) Liver resection for CRC metastasis four years ago. (c) Biopsy of intrabiliary mass. ((d) and (e)) Resection of the intrabiliary metastasis. (f) Smaller liver nodular lesions. All images are H & E (×40).

**Figure 2:**
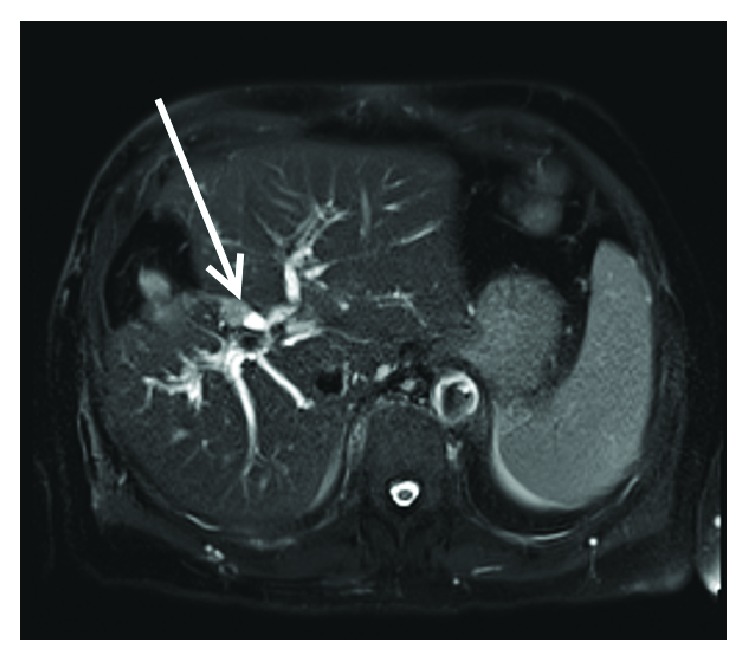
MRI showing the intrahepatic lesion.

A 71-year-old gentleman presented with painless jaundice. His initial laboratory evaluation showed elevated AST, ALT, alkaline phosphatase, serum bilirubin, and CA19.9. His past history was significant for rectosigmoid adenocarcinoma (pT1N0) resection ten years ago (Figure 1(a)). He also had liver resection for colorectal cancer metastasis four years ago (Figure 1(b)) and history of melanoma with resection a year ago. Further evaluation by the MRI study showed a 1.4 × 1.1 cm intraductal mass consistent with primary cholangiocarcinoma at hepatic hilum with severe bilobar intrahepatic ductal dilation (Figure 2). ERCP showed intraductal segmental biliary stricture in the common hepatic duct. A biopsy was done and a stent was placed. The pathologic evaluation of biopsy from the bile duct stricture was significant for adenocarcinoma (Figure 1(c)). Because of papillary architecture and intraductal presence of a mass, a diagnosis of primary cholangiocarcinoma was favored.

**Figure 3:**
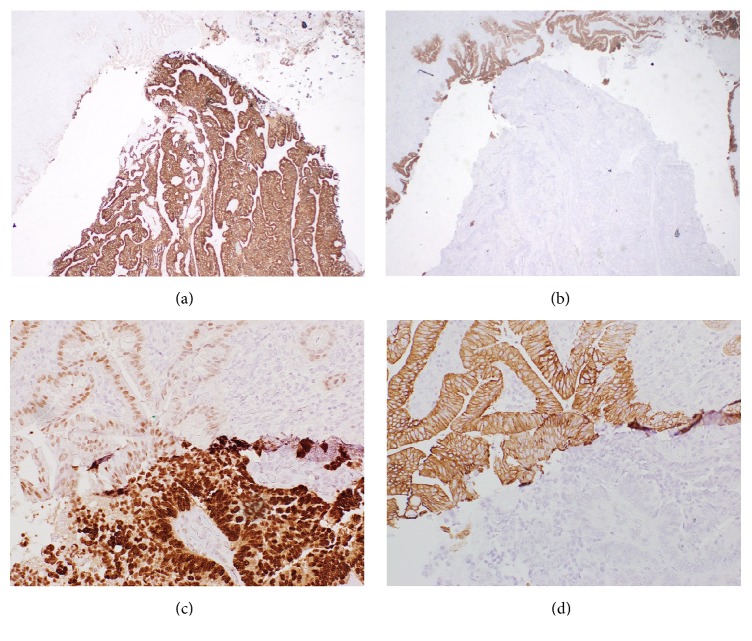
The intrabiliary tumor is positive for CDX2 staining (a and c) and negative for CK7 staining (b and d). CK 7 highlights background bile duct epithelium (b and d). (a and b): ×40; (c and d): ×200.

The patient subsequently underwent central hepatectomy with biliary reconstruction with Roux-en-Y hepaticojejunostomy. On laparotomy, he was found to have a mass at the hilar bifurcation compressing the bile duct. The patient thus underwent resection of segments 4 and 5A and resection of the common bile duct and left and right hepatic ducts. On gross examination, there were multiple tan-white nodules ranging in size from 0.1 to 0.5 mm in the central liver. Within the duct at the bifurcation, there was a polypoid mass into the lumen, measuring 1.2 cm in the greatest dimension. Sections from these sites showed adenocarcinoma. Intrabiliary tumor showed papillary architectures with colonization of bile ducts (Figures 1(d) and 1(e)). There was dilation of bile ducts with intrabiliary tumors. Smaller satellite nodules in liver showed typical metastatic colon carcinoma like morphology (Figure 1(f)). Because of these two different morphological patterns, immunostains were performed. However, IHC staining of both intrabiliary and smaller hepatic nodules demonstrated same pattern with positive CDX2 expression and CK20 expression and negative for CK7 (Figure 3). Based on patient's history and current pathologic findings, a diagnosis was made of metastatic intrabiliary CRLM. The patient finished three cycles of adjuvant chemotherapy. Subsequent surveillance scan was unremarkable. The patient was doing well on surveillance with no new symptoms.

## 3. Discussion

Intrabiliary growth of metastatic carcinoma was first reported in 1946 with symptomatic jaundice, biliary dilatation, and cirrhosis on autopsy [6]. Study from MD Anderson Cancer Center revealed that the primary lesions of intrabiliary growth of hepatic metastasis are composed predominantly of colorectal carcinoma (93%), among which the rectosigmoid colon is the most common site of the primary tumor [2]. According to this study, 72% CRLM show major duct involvement, and 28% show minor duct involvement. It has been reported that CRLM with major bile duct involvement may cause more obvious clinical symptoms, such as abnormal LFTs (liver function tests) indicating the biliary obstruction, abnormal imaging results that show biliary disease, and histologic findings related to biliary disease and secondary sclerosing cholangitis. Rare cases demonstrate the intrahepatic tumor dissemination and multiple tumor nodules.

Two patterns of intrabiliary metastasis are described: colonization of the bile duct and tumor floating and blocking the ductal lumen, with each pattern comprising 81% of the cases [2]. Our report fits more colonization pattern. There are controversial opinions on the prognosis of the different patterns of CRLM. Okano et al. found that patients with macroscopic bile duct invasion obtain the highest 5-year survival rate compared with patient showing microscopic bile duct invasion and no bile duct invasion [7]. Kubo et al. explained this paradoxical finding that the tumor with macroscopic biliary involvement is less aggressive and better differentiated [8]. Nevertheless, studies performed by Povoski et al. and Estrella et al. both indicated that there are actually no significant differences of survival rate and differentiation between major and minor duct involvement groups [9]. In our case, we found that the metastatic adenocarcinomas involved predominantly major duct and produce tumor nodules with smaller hepatic metastasis, however, with rare minor duct involvement, as well. The intrabiliary metastatic lesion retains some features of the primary lesion, such as cribriform architecture, stratified, pencil-shaped nuclei, and dirty necrosis. However, it had some morphologic aspects that mimic the PICC, which is usually moderate to well-differentiated adenocarcinoma with glandular and tubular formation [10]. These mimics can be even complicated by the laboratory and imaging similarity between these two types of lesions [3, 9]. Based on the mechanism and pathophysiologic changes, the LFTs abnormalities related to biliary obstruction such as elevated CEAs and alkaline phosphatase are commonly found in CRLM. The CT, MRI, and/or ERCP findings of CRLM are usually nonspecific, comprising intrahepatic mass, biliary dilatation, and/or mural thickening [11, 12].

Therefore, differentiation of the CRLM from the PICC relies on the careful scrutinizing of the patient's clinical history and the application of IHC studies. Although most of the metastasis happens within 3 years from the discovery and resection of the primary lesion, the metastasis in our case happens almost 10 years after the treatment of the primary resection [2]. Such long interval between the intrahepatic lesion and the primary tumor may mask the disease progression. This extremely difficult diagnosis can be established with CD20−/CK7+ staining. Although CK20 is low to moderate specific for PICC, the combination of CK20 and CK7 can reliably differentiate PICC from CRLM [10, 13, 14].

In conclusion, we reported a case of intrabiliary CRLM and uncommon metastatic lesion discovered 10 years after the excision of the primary colorectal adenocarcinoma, as one of the first reports of imaging and serial pathology studies during long term follow-up. Due to the rarity of these metastases in the United States, long intervals after the excision of primary lesion, and its similarity with PICC in radiologic and morphologic evaluation, CRLM can be misdiagnosed as PICC even with major duct involvement, as seen in this patient. Therefore, the past medical history of colorectal carcinoma and application of IHC studies of CK7, CK20, and CDX2 are helpful to achieve the correct diagnosis and management.
